# Spatial Domain Terahertz Image Reconstruction Based on Dual Sparsity Constraints

**DOI:** 10.3390/s21124116

**Published:** 2021-06-15

**Authors:** Xiaozhen Ren, Yuying Jiang

**Affiliations:** 1 School of Artificial Intelligence and Big Data, Henan University of Technology, Zhengzhou 450001, China; yyjing21@126.com; 2 Key Laboratory of Grain Information Processing & Control, Ministry of Education, Henan University of Technology, Zhengzhou 450001, China

**Keywords:** terahertz, imaging model, exponentiation shift invariant wavelet, gradient domain, split Bregman iteration

## Abstract

Terahertz time domain spectroscopy imaging systems suffer from the problems of long image acquisition time and massive data processing. Reducing the sampling rate will lead to the degradation of the imaging reconstruction quality. To solve this issue, a novel terahertz imaging model, named the dual sparsity constraints terahertz image reconstruction model (DSC-THz), is proposed in this paper. DSC-THz fuses the sparsity constraints of the terahertz image in wavelet and gradient domains into the terahertz image reconstruction model. Differing from the conventional wavelet transform, we introduce a non-linear exponentiation transform into the shift invariant wavelet coefficients, which can amplify the significant coefficients and suppress the small ones. Simultaneously, the sparsity of the terahertz image in gradient domain is used to enhance the sparsity of the image, which has the advantage of edge preserving property. The split Bregman iteration scheme is utilized to tackle the optimization problem. By using the idea of separation of variables, the optimization problem is decomposed into subproblems to solve. Compared with the conventional single sparsity constraint terahertz image reconstruction model, the experiments verified that the proposed approach can achieve higher terahertz image reconstruction quality at low sampling rates.

## 1. Introduction

Terahertz band refers to the electromagnetic spectrum region with frequency from 100 GHz to 10 THz, which is between millimeter wave and infrared light. Terahertz radiation has unique characteristics and applications in the field of imaging due to its perspective of many optically opaque materials, low-energy lossless and spectral resolution [1,2,3]. Therefore, terahertz imaging technology has great application potential in many fields, such as nondestructive testing, identification of hidden materials and food production quality monitoring [4,5,6,7,8]. In particular, terahertz radiation could recognize biomolecules and its photon energy is too low to cause atoms ionization, so it is attractive for noninvasive biomedical imaging [9,10,11]. In the field of biomedical imaging, the image quality is the most important standard. High-quality images require high resolution and high signal-to-noise ratio, which can be easily obtained by terahertz time domain spectroscopy (THz-TDS) imaging systems.

THz-TDS imaging is one of the simplest terahertz imaging modes. It can acquire high-quality object images with high spatial resolution by raster scanning. However, THz-TDS technology requires space scanning to perform imaging, which has the problems of long image acquisition time and massive data processing. The fast-imaging techniques of THz-TDS are desirable for its practical application. To reduce the long image acquisition time by raster scanning, various techniques for THz-TDS fast imaging have been proposed. Array detectors have been used to overcome the image acquisition speed limitations of sequential data acquisition [12,13]. However, these methods have higher complexity and operational cost. A fast-pulsed terahertz Fourier imaging method based on compressed sensing (CS) was proposed in [14], which allows image reconstruction with a lower sample rate than the traditional method required by utilizing the image sparsity in the frequency domain. However, the image acquisition speed of this method is still limited because the detector is performing a raster scan in the Fourier plane. Subsequently, a single pixel terahertz imaging system based on CS was proposed [15,16]. This system does not require mechanical scanning of the terahertz receiver on the image plane. The spatial profile of the terahertz beam passing the object is modulated by a random pattern, and the resulting beam is focused by the lens onto the single fixed detector. Then, by changing the random patterns, data corresponding to the different patterns are collected, and the image can be reconstructed by CS. Although the number of measurements used for image reconstruction is much smaller than the traditional raster scan system, an additional problem is that this method has slow translation of one random pattern to another. Although other spatial modulation schemes of a terahertz beam driven by optically or electrically were researched [17,18], this method still requires additional hardware devices for the spatial modulation.

In addition, fast spatial domain terahertz imaging using block-based CS was proposed in [19,20]. This method can shorten scan time and speed up the imaging processing of the conventional terahertz imaging systems without any hardware addition or modification. However, this method only uses the sparsity of the terahertz image in the frequency domain for reconstruction, and the reconstruction quality is degraded when the sampling rate is reduced. How to further improve the terahertz image reconstruction quality by exploiting more prior information of the image will be a crucial issue. In order to solve this issue, a novel terahertz imaging method from undersampled data which fuses the dual sparsity constraints of the terahertz image in wavelet and gradient domains is proposed in this paper. To enhance the sparsity of the terahertz image in the wavelet domain, a non-linear exponentiation transform is introduced into the shift invariant wavelet coefficients, which can amplify the significant coefficients and suppress the small ones. Simultaneously, the sparsity of the terahertz image in gradient domain is used to enhance the sparsity of the image for ensuring high-quality image reconstruction, which has the advantage of edge preserving property [21,22]. Therefore, terahertz image can be accurately reconstructed from the undersampled data.

The rest of the paper is organized as follows. Section 2 presents the spatial domain signal model of THz-TDS system. In Section 3, a novel terahertz imaging method that fuses the dual sparsity constraints of the terahertz image in wavelet and gradient domains is described in detail. The performance of the proposed method is investigated in Section 4. Section 5 gives a brief conclusion.

## 2. Spatial Domain Signal Model of THz-TDS System

Suppose that the sample image ***x*** has *m* × *n* pixels. Let *N* = *m* × *n*, and randomly select the *M* positions from the *N* pixels for terahertz detection; then, a complete terahertz time domain waveform data will be obtained at each detection position in THz-TDS system. Select the peak value of each time domain waveform as the pixel value of the corresponding detection position; then, the sparse terahertz imaging system model in the spatial domain can be expressed as

(1) y=Rx

where ***y*** is the *M* dimensional terahertz measured data. ***x*** is the sample image, and its *m* × *n* elements are arranged in an *N* dimensional column vector. **R** is an *M* × *N* measurement matrix with only one element equal to 1 and the others are equal to 0 in each row, and the positions of the elements with the value of 1 are determined by the detection positions.

In conclusion, the objective of the terahertz imaging is to reconstruct the sample image ***x*** from the sparse measured data ***y***. As the spectral density of an ordinary terahertz image is usually distributed in a low-frequency band, representing strong sparsity, the terahertz imaging can be transformed into the problem of sparse signal reconstruction, and the CS-based method can be used for terahertz image reconstruction [23]. Among the various frequency domain transform methods, wavelet transform has good spatial and frequency characteristics, and it is often used as the sparse transform for image reconstruction. By utilizing the sparsity of the terahertz image in the wavelet transform domain, the terahertz image can be reconstructed by

(2) $$\begin{array}{l} \underset{x}{\min}\left\{{\left\|Fx\right\|}_{1}\right\}\;\\ s.t.\;y=Rx \end{array}$$

where *F* denotes the discrete wavelet transform.

## 3. The Proposed DSC-THz Imaging Method

### 3.1. Proposed DSC-THz Model

In order to further improve the terahertz image reconstruction quality, a novel DSC-THz model based on the dual sparsity constraints of the terahertz image in wavelet and gradient domains is proposed in this paper. The orthogonal wavelet basis is a set of functions obtained by the basic wavelet function through translation and stretching transform. With the increase in scale, the displacement sampling interval increases with the power of 2, which could not match the local structure characteristics of the signal from the multi-scale. Therefore, oscillation and block effect may occur in the region where the signal changes sharply. In order to effectively eliminate this artificial oscillation phenomenon, the shift invariant wavelet transform is used in this paper to decompose the terahertz image, and the sparse representation of the terahertz image is obtained by

(3) $$r=W(x)=\Psi^{-1}x$$

where *W*(***x***) denotes the shift invariant wavelet transform of the image ***x***, Ψ denotes the shift invariant wavelet transform matrix, and ***r*** is the shift invariant wavelet coefficient.

**Figure 1:**
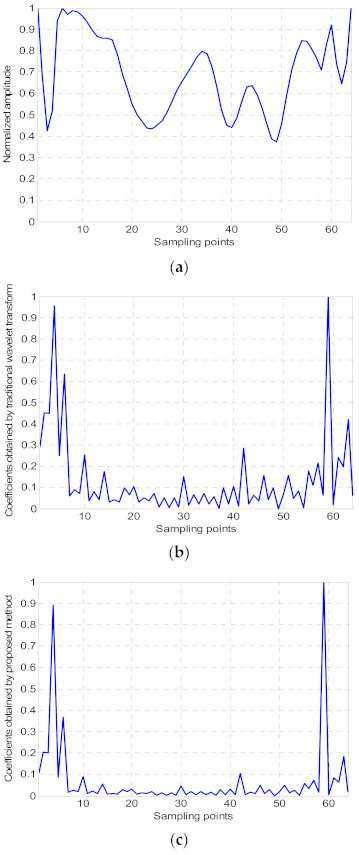
Comparison of the wavelet coefficients obtained by traditional wavelet transform and proposed method. (**a**) Original one-dimensional signal; (**b**) wavelet coefficients obtained by traditional wavelet transform; (**c**) wavelet coefficients obtained by proposed method.

The performance of CS depends on the sparsity of the image in the sparse domain. To enhance the sparsity, the exponentiation transform is introduced into the wavelet coefficients and has been proven to be more efficient in sparse representation [24]. Inspired by exponentiation transform [24], we transformed the shift invariant wavelet coefficients via the non-linear exponential function to enhance the sparsity of the terahertz images in the wavelet transform domain, which could amplify the significant coefficients and suppress the small ones. The proposed exponential shift invariant wavelet coefficients can be written as:

(4) $$r_{e}=W_{e}x=\frac{a^{r}-1}{a-1}=\frac{a^{\Psi^{-1}x}-1}{a-1}$$

where $W_{e}x$ denotes the exponential shift invariant wavelet transform of the image ***x***, Ψ denotes the shift invariant wavelet transform matrix, and the wavelet coefficients are normalized here. *a* is a constant greater than 1, which is set to 10 in this paper. Figure 1 gives a simple example to compare the wavelet coefficients obtained by traditional wavelet transform and the proposed method. Figure 1a is a one-dimensional signal taken from a line of the two-dimensional terahertz image. Figure 1b,c are the wavelet coefficients obtained by traditional wavelet transform and the proposed method, respectively. From Figure 1, it is clear that the proposed method could further enhance the sparsity of the wavelet coefficients.

Furthermore, in order to improve the reconstruction quality of the terahertz image, we also exploit the sparsity of the terahertz image in gradient domain to enhance the sparsity, which has the advantage of edge preserving property [21,22]. The gradient image of the terahertz image is obtained as

(5) $$x_{t}=Tx=\left|T_{x}x\right|+\left|T_{y}x\right|$$

where *T**x*** represents gradient operation on the image ***x***. $T_{x}$ and $T_{y}$ denote the gradient operators on the horizontal and vertical directions, respectively.

We obtain

(6) $$T_{x}x=\mathrm{vec}(\nabla x^{x}(m,n))$$

(7) $$T_{y}x=\mathrm{vec}(\nabla x^{y}(m,n))$$

(8) $$\nabla x^{x}(m,n)=\left\{\begin{array}{c} x(m,n)\;\;\;\;\;\;\;n=1\\ x(m,n)-x(m,n-1)\;1<n\leq N \end{array}\right.$$

(9) $$\nabla x^{y}(m,n)=\left\{\begin{array}{c} x(m,n)\;\;\;\;\;\;\;m=1\\ x(m,n)-x(m-1,n)\;1<m\leq M \end{array}\right.$$

In conclusion, combined with the sparsity constraints of the terahertz image in wavelet and gradient domains, the proposed DSC-THz model can be expressed as

(10) $$\begin{array}{l} \underset{x}{\min}\left\{{\left\|W_{e}x\right\|}_{1}+{\left\|T_{x}x\right\|}_{1}+{\left\|T_{y}x\right\|}_{1}\right\}\;\\ s.t.\;y=Rx \end{array}$$

To solve the optimization problem (10), we convert it into an unconstrained optimization problem by adding the penalty function term

(11) $$\underset{x,r_{e},d_{x},d_{y}}{\min}{\left\|W_{e}x\right\|}_{1}+{\left\|T_{x}x\right\|}_{1}+{\left\|T_{y}x\right\|}_{1}+\frac{\mu }{2}{\left\|Rx-y\right\|}_{2}^{2}$$

where μ is regularization parameter. As the gradient operator is nonsmooth in ***x***, it is very difficult to solve the optimization problem (11) involving multiple *l*_1_-norm terms.

### 3.2. The Proposed Algorithm

Split Bregman iteration is a method that originated in functional analysis for finding extrema of convex functionals, which can split a complex optimization problem into a small number of unconstrained subproblems to solve. Moreover, an advantage of split Bregman iteration is that the value of the regularization parameters could remain constant in the iterations, resulting in fast convergence for the optimization method [25,26,27].

By applying the split Bregman iteration scheme to our imaging method, we first define $r_{e}=W_{e}x$, $d_{x}=T_{x}x$ and $d_{y}=T_{y}x$; then, the split Bregman formulation of the optimization problem (11) becomes

(12) $$\begin{array}{l} \left(x^{i+1},r_{e}^{i+1},d_{x}^{i+1},d_{y}^{i+1}\right)=\underset{x,r_{e},d_{x},d_{y}}{\min}{\left\|r_{e}\right\|}_{1}+{\left\|d_{x}\right\|}_{1}+{\left\|d_{y}\right\|}_{1}+\frac{\mu }{2}{\left\|Rx-y\right\|}_{2}^{2}+\frac{\lambda }{2}{\left\|r_{e}^{}-W_{e}x-b_{r}^{i}\right\|}_{2}^{2}\\ \;\;\;\;\;\;\;\;\;\;\;+\frac{\gamma }{2}{\left\|d_{x}^{}-T_{x}x-b_{x}^{i}\right\|}_{2}^{2}+\frac{\gamma }{2}{\left\|d_{y}^{}-T_{y}x-b_{y}^{i}\right\|}_{2}^{2} \end{array}$$

where λ and γ are regularization parameters, and

(13) $$b_{r}^{i+1}=b_{r}^{i}+(W_{e}x^{i+1}-r_{e}^{i+1})$$

(14) $$b_{x}^{i+1}=b_{x}^{i}+(T_{x}x^{i+1}-d_{x}^{i+1})$$

(15) $$b_{y}^{i+1}=b_{y}^{i}+(T_{y}x^{i+1}-d_{y}^{i+1})$$

Using the idea of separation of variables, the optimization problem (12) can be decomposed into four unconstrained optimization subproblems, as follows:

Subproblem 1: Solving the ***x*** subproblem

Fixing ***r****_e_*, ***d****_x_*, ***d****_y_*, ***b****_r_*, ***b****_x_* and ***b****_y_*, the optimization function of the ***x*** is derived by splitting (12)

(16) $$\begin{array}{l} x^{i+1}=\underset{x}{\min}\frac{\mu }{2}{\left\|Rx-y\right\|}_{2}^{2}+\frac{\lambda }{2}{\left\|r_{e}^{i}-W_{e}x-b_{r}^{i}\right\|}_{2}^{2}\\ \;\;\;\;+\frac{\gamma }{2}{\left\|d_{x}^{i}-T_{x}x-b_{x}^{i}\right\|}_{2}^{2}+\frac{\gamma }{2}{\left\|d_{y}^{i}-T_{y}x-b_{y}^{i}\right\|}_{2}^{2} \end{array}$$

As we have decoupled ***x*** from the *l*_1_ portion of the optimization problem (12), the subproblem (16) that we must solve for ***x*** is now differentiable, and optimality conditions for ***x*** are easily derived. By differentiating with respect to ***x*** and setting the result equal to zero, we get the update rule

(17) $$\begin{array}{l} \left(\mu R^{T}R+\gamma T_{x}^{T}T_{x}+\gamma T_{y}^{T}T_{y}+\lambda I\right)x^{i+1}\\ =\mu R^{T}y+\gamma T_{x}^{T}\left(d_{x}^{i}-b_{x}^{i}\right)+\gamma T_{y}^{T}\left(d_{y}^{i}-b_{y}^{i}\right)+\lambda W_{e}^{T}\left(r_{e}^{i}-b_{r}^{i}\right) \end{array}$$

Set

(18) $$z^{i}=\mu R^{T}y+\gamma T_{x}^{T}\left(d_{x}^{i}-b_{x}^{i}\right)+\gamma T_{y}^{T}\left(d_{y}^{i}-b_{y}^{i}\right)+\lambda W_{e}^{T}\left(r_{e}^{i}-b_{r}^{i}\right)$$

Then

(19) $$x^{i+1}={\left(\mu R^{T}R+\gamma T_{x}^{T}T_{x}+\gamma T_{y}^{T}T_{y}+\lambda I\right)}^{-1}z^{i}$$

Subproblem 2: Solving the ***r****_e_* subproblem

Fixing ***x***, ***d****_x_*, ***d****_y_*, ***b****_r_*, ***b****_x_* and ***b****_y_*, the optimization function of the ***r****_e_* is derived by splitting (12)

(20) $$r_{e}^{i+1}=\underset{r_{e}}{\min}{\left\|r_{e}\right\|}_{1}+\frac{\lambda }{2}{\left\|r_{e}-W_{e}x^{i+1}-b_{r}^{i}\right\|}_{2}^{2}\;$$

which can be effectively solved by the shrinkage operator [28,29,30]

(21) $$r_{e}^{i+1}=\mathrm{shrink}\left(W_{e}x^{i+1}+b_{r}^{i},1/\lambda \right)$$

where

(22) $$\mathrm{shrink}\left(x,\lambda \right)=\frac{x}{\left|x\right|}\times \max\left(\left|x\right|-\lambda ,0\right)$$

Subproblem 3: Solving the ***d****_x_* subproblem

Fixing ***x***, ***r****_e_*, ***d****_y_*, ***b****_r_*, ***b****_x_* and ***b****_y_*, the optimization function of the ***d****_x_* is derived by splitting (12)

(23) $$d_{x}^{i+1}=\underset{d_{x}}{\min}{\left\|d_{x}\right\|}_{1}+\frac{\gamma }{2}{\left\|d_{x}-T_{x}x^{i+1}-b_{x}^{i}\right\|}_{2}^{2}\;$$

which can be effectively solved by the shrinkage operator

(24) $$d_{x}^{i+1}=\mathrm{shrink}\left(T_{x}x^{i+1}+b_{x}^{i},1/\gamma \right)$$

Subproblem 4: Solving the ***d****_y_* subproblem

Fixing ***x***, ***r****_e_*, ***d****_x_*, ***b****_r_*, ***b****_x_* and ***b****_y_*, the optimization function of the ***d****_y_* is derived by splitting (12)

(25) $$d_{y}^{i+1}=\underset{d_{y}}{\min}{\left\|d_{y}\right\|}_{1}+\frac{\gamma }{2}{\left\|d_{y}-T_{y}x^{i+1}-b_{y}^{i}\right\|}_{2}^{2}\;$$

which can be effectively solved by the shrinkage operator

(26) $$d_{y}^{i+1}=\mathrm{shrink}\left(T_{y}x^{i+1}+b_{y}^{i},1/\gamma \right)$$

**Figure 2:**
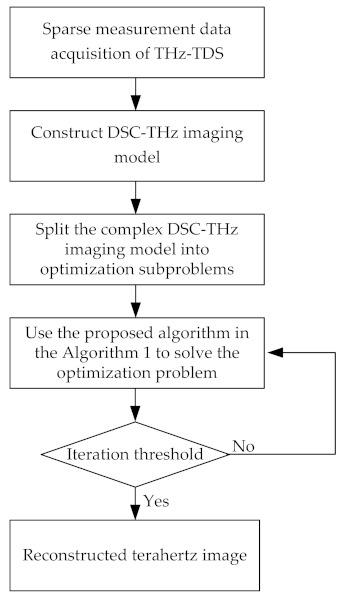
The processing flow of the proposed spatial domain terahertz image reconstruction method.

The main steps of the proposed algorithm are summarized in the Algorithm 1. Additionally, the whole processing flow of the proposed spatial domain terahertz image reconstruction method is shown in Figure 2.
**Algorithm 1.** Proposed DSC-THz Imaging Algorithm**Input:** measurement ***y***, measurement matrix **R**, exponential shift invariant wavelet basis ***W****_e_*, horizontal gradient operator $T_{x}$, vertical gradient operator $T_{y}$. **Initialization:** $x^{0}=R^{-1}y$,$r_{e}^{0}=d_{x}^{0}=d_{y}^{0}=b_{r}^{0}=b_{x}^{0}=b_{y}^{0}=0$,μ,λ,γ **Loop:** set i=0 and repeat until ($||x^{i+1}-x^{i}|{|}_{2}<\delta$)   $x^{i+1}={\left(\mu R^{T}R+\gamma T_{x}^{T}T_{x}+\gamma T_{y}^{T}T_{y}+\lambda I\right)}^{-1}z^{i}$
  $r_{e}^{i+1}=\mathrm{shrink}\left(W_{e}x^{i+1}+b_{r}^{i},1/\lambda \right)$
  $d_{x}^{i+1}=\mathrm{shrink}\left(T_{x}x^{i+1}+b_{x}^{i},1/\gamma \right)$
  $d_{y}^{i+1}=\mathrm{shrink}\left(T_{y}x^{i+1}+b_{y}^{i},1/\gamma \right)$
  $b_{r}^{i+1}=b_{r}^{i}+(W_{e}x^{i+1}-r_{e}^{i+1})$
  $b_{x}^{i+1}=b_{x}^{i}+(T_{x}x^{i+1}-d_{x}^{i+1})$
  $b_{y}^{i+1}=b_{y}^{i}+(T_{y}x^{i+1}-d_{y}^{i+1})$
  i=i+1
**End loop**
**Output:** reconstructed terahertz image ***x***.

### 3.3. Convergence Analysis

The convergence of Algorithm 1 is described as Theorem 1, and the proof of Theorem 1 is shown in the Appendix A.

**Theorem** **1.** *Assume that there exists one solution* $x^{*}$ *to the optimization problem (11). We then have*

(27) $$\begin{array}{l} \underset{i\to \infty }{\lim}(||W_{e}x_{}^{i}|{|}_{1}+||T_{x}x_{}^{i}|{|}_{1}+||T_{y}x_{}^{i}|{|}_{1}+\frac{\mu }{2}||Rx^{i}-y|{|}_{2}^{2})\\ \;\;=||W_{e}x^{*}|{|}_{1}+||T_{x}x^{*}|{|}_{1}+||T_{y}x^{*}|{|}_{1}+\frac{\mu }{2}||Rx^{*}-y|{|}_{2}^{2} \end{array}$$

## 4. Experiments and Discussion

In this section, real terahertz data were processed to verify the performance of the proposed DSC-THz model in practice.

**Figure 3:**
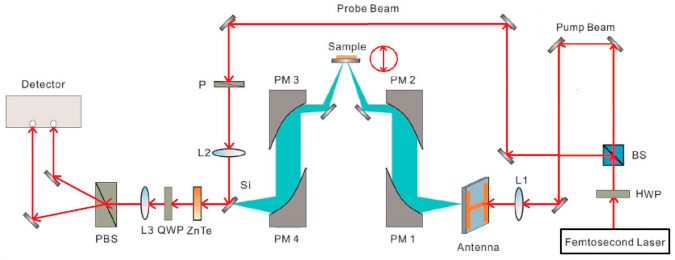
THz-TDS reflection imaging system.

A standard THz-TDS laboratory setup, using reflection geometry developed by Zomega terahertz company in USA, was used in our experiment. The measurement range of this system is 5 cm × 5 cm. A typical THz-TDS reflection imaging system is shown in Figure 3. The pulsed terahertz beam driven by a femtosecond laser Ti-sapphire has a central wavelength and pulse width of 800 nm and 100 fs, respectively. The beam passes through a half wave plate and then is split into pump and probe beams by a beam splitter. The half wave plate is used to adjust the beam splitter to change the intensity of the two separate beams. The pump beam is irradiated on a photoconductive switch fabricated on a LT-GaAs wafer for generation of the terahertz waves, and the probe beam is focused onto an electro-optic ZnTe crystal for detection of the of terahertz waves [31,32,33]. The terahertz pulse emitted from the generator is focused on the sample by two metal parabolic mirrors. It is then reflected by the sample via two additional parabolic mirrors and guided to the ZnTe crystal, where it is overlapped with the probe beam. The probe beam is modulated by the terahertz field within the ZnTe crystal [34,35]. The modulated probe beam then passes through a quarter wave plate to make the phase difference π/2 between o-light and e-light, and then it is divided into two beams with mutually perpendicular polarization directions by polarization beam splitter to incident on the detector. The sample moves in a raster scanning mode, and the experiment is implemented at room temperature.

The proposed sparse terahertz imaging system can be easily implemented from the conventional THz-TDS imaging system by programming the scanner to move according to the sampling positions defined by the measurement matrix. In this experiment, the sparse measurement data are obtained from the raster scan according to the sampling positions of the measurement matrix. Let *N* denote the number of pixels of the sample image, *M* denote the number of the pixels scanned, *P* denote the time taken to scan a pixel, and *Q* be the total time taken to move the sample in the raster scanning mode. The whole imaging time of the proposed sparse terahertz imaging system can be written as *MP + Q*, and *M = N* for the conventional THz-TDS imaging system. Therefore, under the certain system parameters, the imaging time is determined by the sampling rate *M*/*N*. Fast imaging can be achieved by reducing *M*.

**Figure 4:**
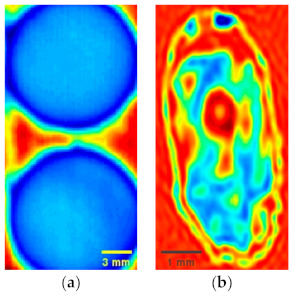
The full scan terahertz images of the two samples. (**a**) Circular solids made from wheat flour; (**b**) wheat seed.

In order to evaluate the performance of our algorithm, two samples are used in the experiment. Sample 1 contains two circular solids made from wheat flour under 10 MPa pressure. The thickness of the circular solid is 1.2 mm, and the diameter is 13 mm. The experimental humidity is 22%, and the experimental temperature is 24 °C. Sample 2 is a wheat seed. The experimental humidity is 15%, and the experimental temperature is 25 °C. The samples move in a raster scanning mode, and the full scan terahertz images for the two samples are shown in Figure 4.

In addition to characterizing the performance quantitatively, the peak signal to noise ratio (PSNR) is used as the evaluation for the reconstruction quality of the terahertz image. The PSNR is defined as [19]

(28) $$PSNR=10\log_{10}\frac{peakval^{2}}{MSE(x,\hat{x})}$$

where *peakval* is the maximum value of the image. $MSE(x,\hat{x})$ is the mean squared error between the true image *x* and the estimated image $\hat{x}$.

**Figure 5:**
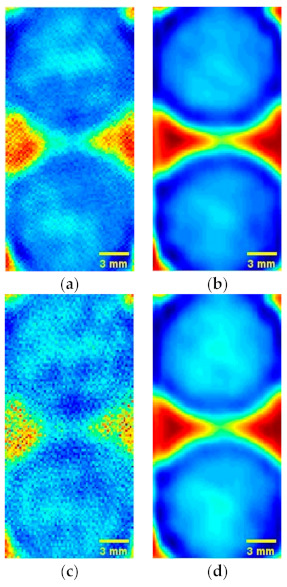
Reconstructed results comparation of DSC-THz and SSC-THz with different sampling rates for the circular solids. (**a**) DSC-THz with sampling rate 10%; (**b**) DSC-THz with sampling rate 30%; (**c**) SSC-THz with sampling rate 10%; (**d**) SSC-THz with sampling rate 30%.

In order to analyze the performance of the proposed DSC-THz, conventional single sparsity constraint terahertz image reconstruction model (SSC-THz) [20] is given for comparison. Figure 5 shows the reconstructed terahertz images of the circular solids made from wheat flour at different sampling rates. Figure 5a,b show the reconstruction results using the proposed DSC-THz at sampling rates of 10% and 30%, respectively. Additionally, Figure 5c,d present the reconstruction results obtained by the conventional SSC-THz. Compared with the reconstructed images obtained by SSC-THz, the proposed method has obvious advantages. As seen in Figure 5, the reconstructed image obtained by the proposed method has the perceptually equivalent quality to that achieved using full scan at the sampling rate of 30%. When the sampling rate decreases, the image quality decays. In particular, the reconstructed image obtained by SSC-THz is severely degraded at the sampling rate of 10%.

**Figure 6:**
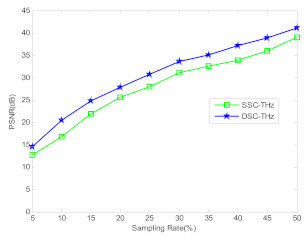
Comparison of the PSNR to different sampling rates.

In order to illustrate the superiority property of the proposed method, Figure 6 shows the PSNR curves of DSC-THz and SSC-THz changing with the different sampling rates for the terahertz image of circular solids. It can be seen from Figure 6 that the proposed DSC-THz provides larger PSNR value compared to SSC-THz, which means the proposed method has better reconstruction performance.

**Figure 7:**
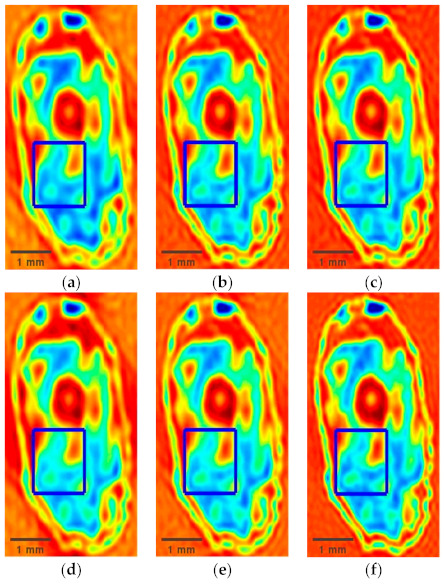
Reconstructed results comparation of DSC-THz and SSC-THz with different sampling rates for the wheat seed. (**a**) DSC-THz with sampling rate 20%; (**b**) DSC-THz with sampling rate 30%; (**c**) DSC-THz with sampling rate 40%; (**d**) SSC-THz with sampling rate 20%; (**e**) SSC-THz with sampling rate 30%; (**f**) SSC-THz with sampling rate 40%.

**Figure 8:**
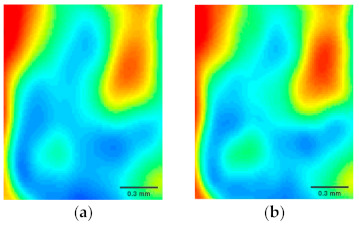

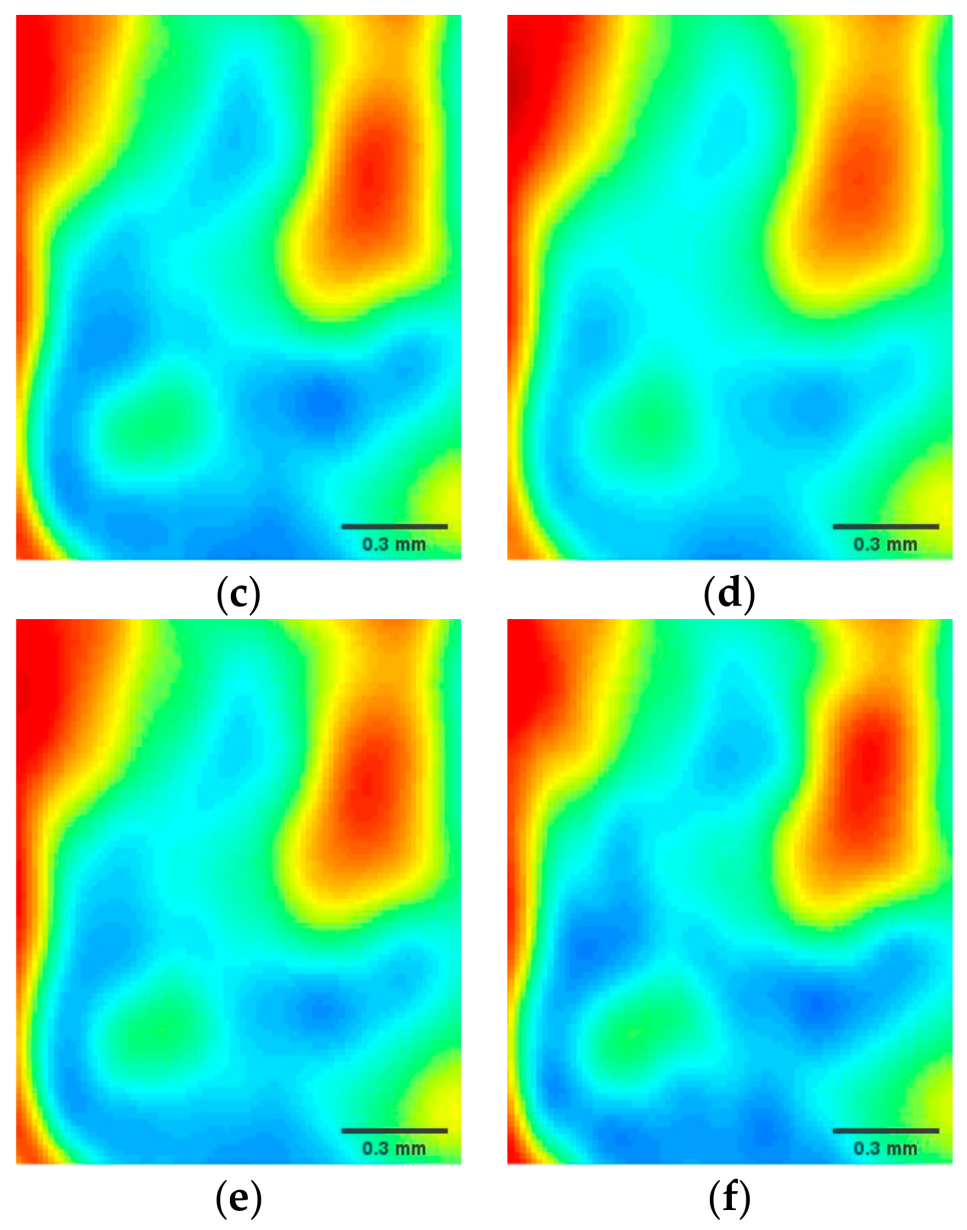
Reconstructed results comparation of DSC-THz and SSC-THz with different sampling rates at the selected regions. (**a**) DSC-THz with sampling rate 20%; (**b**) DSC-THz with sampling rate 30%; (**c**) DSC-THz with sampling rate 40%; (**d**) SSC-THz with sampling rate 20%; (**e**) SSC-THz with sampling rate 30%; (**f**) SSC-THz with sampling rate 40%.

Applying the proposed DSC-THz to the terahertz imaging of wheat seed is shown in Figure 7. Figure 7a–c show the reconstruction results using DSC-THz with different sampling rates from 20% to 40%, respectively. Additionally, Figure 7d–f present the reconstruction results obtained by SSC-THz. It can be seen that the reconstructed images obtained by DSC-THz appear similar to that by full scan at the sampling rates of 30% and 40%. However, some degradations are observed in the reconstructed images obtained by SSC-THz at the same sampling rates. Moreover, when the sampling rate decreases to 20%, DSC-THz still has better reconstruction capacity, as can be seen in Figure 7a,d. It also can be seen from Figure 7 that there are some partial losses in the reconstruction images details by using SSC-THz, while the proposed method can better reconstruct the image and preserve more image details. For a close-up comparison, we enlarged the selected regions with the red rectangles in Figure 8 to evaluate the image quality. Figure 8 presents the reconstruction results of the selected regions. From Figure 8, it is obvious that the reconstructed images obtained by SSC-THz lost some detail information of the images.

**Figure 9:**
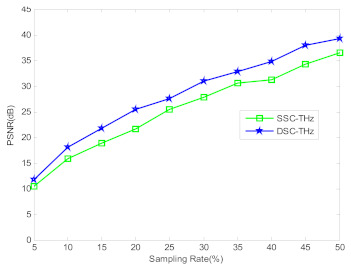
Comparison of the PSNR to different sampling rates.

To further illustrate the performance of DSC-THz, Figure 9 shows the PSNR curves of DSC-THz and SSC-THz changing with the different sampling rates for the terahertz image of wheat seed. As seen in Figure 9, DSC-THz provides higher PSNR compared to SSC-THz. Compared with Figure 6 and Figure 9, it can be seen that the PSNR of the wheat seed image is a little lower than that of circular solids in low sampling rates, that is because the image of wheat has a more complex structure than the circular solids. Overall, DSC-THz is able to reconstruct images with better PSNR than SSC-THz.

The above results demonstrated that simultaneously using the sparsity constraints of the terahertz image in wavelet and gradient domains can achieve better reconstruction results.

## 5. Conclusions

THz-TDS imaging technology has been used widely in many fields such as nondestructive testing, medical imaging and food production quality monitoring. However, THz-TDS imaging systems suffer from long image acquisition time and massive data processing because of their raster-scanning mechanism. A novel DSC-THz model based on the dual sparsity constraints has been proposed to effectively reconstruct terahertz image from undersampled data in this paper. The sparsity constraints of the terahertz image in wavelet and gradient domains are applied to the proposed model simultaneously, whose advantage is that it could enhance the sparsity and has better edge preserving property. Furthermore, we employ the split Bregman iteration scheme to tackle the optimization problem effectively. By using the idea of separation of variables, the optimization problem of DSC-THz can be decomposed into a series of subproblems to solve. Various experiment results with the real data confirm that the proposed method has the superior performance for terahertz image reconstruction. How to further achieve fast and accurate terahertz image reconstruction under lower sampling rate is the next focus of our research work.

## Data Availability

The data presented in this study are available on request from the corresponding author.

## Appendix A

**Proof** **of** **Theorem** **1.** The first order optimality condition of Algorithm 1 yields that

(A1) $$\{\begin{array}{c} \begin{array}{l} \mu R^{T}(Rx^{i+1}-y)+\lambda W_{e}^{T}(W_{e}x^{i+1}-r_{e}^{i}+b_{r}^{i})\\ \;+\gamma T_{x}^{T}\left(T_{x}x^{i+1}-d_{x}^{i}+b_{x}^{i}\right)+\gamma T_{y}^{T}\left(T_{y}x^{i+1}-d_{y}^{i}+b_{y}^{i}\right)=0 \end{array}\\ J_{1}^{i+1}+\lambda (r_{e}^{i+1}-W_{e}x^{i+1}-b_{r}^{i})=0\\ J_{2}^{i+1}+\gamma (d_{x}^{i+1}-T_{x}x^{i+1}-b_{x}^{i})=0\\ J_{3}^{i+1}+\gamma (d_{y}^{i+1}-T_{y}x^{i+1}-b_{y}^{i})=0\\ b_{r}^{i+1}=b_{r}^{i}+(W_{e}x^{i+1}-r_{e}^{i+1})\\ b_{x}^{i+1}=b_{x}^{i}+(T_{x}x^{i+1}-d_{x}^{i+1})\\ b_{y}^{i+1}=b_{y}^{i}+(T_{y}x^{i+1}-d_{y}^{i+1}) \end{array}$$

where $J_{1}^{i+1}=\partial ||r_{e}^{i+1}|{|}_{1}$,$J_{2}^{i+1}=\partial ||d_{x}^{i+1}|{|}_{1}$,$J_{3}^{i+1}=\partial ||d_{y}^{i+1}|{|}_{1}$, and ∂ denotes the subdifferential. By Theorem 1, $x^{*}$ is the solution of the optimization problem (11), so $x^{*}$ satisfies

(A2) $$W_{e}^{T}J_{1}^{*}+T_{x}^{T}J_{2}^{*}+T_{y}^{T}J_{3}^{*}+\mu R^{T}(Rx^{*}-y)=0$$

where $J_{1}^{*}=\partial ||r_{e}^{*}|{|}_{1}$, $J_{2}^{*}=\partial ||d_{x}^{*}|{|}_{1}$ and $J_{3}^{*}=\partial ||d_{y}^{*}|{|}_{1}$. Let $J_{1}^{*}=\lambda b_{r}^{*}$, $J_{2}^{*}=\gamma b_{x}^{*}$ and $J_{3}^{*}=\gamma b_{y}^{*}$, then (A2) is equal to

(A3) $$\{\begin{array}{c} \begin{array}{l} \mu R^{T}(Rx^{*}-y)+\lambda W_{e}^{T}(W_{e}x^{*}-r_{e}^{*}+b_{r}^{*})\\ \;+\gamma T_{x}^{T}\left(T_{x}x^{*}-d_{x}^{*}+b_{x}^{*}\right)+\gamma T_{y}^{T}\left(T_{y}x^{*}-d_{y}^{*}+b_{y}^{*}\right)=0 \end{array}\\ J_{1}^{*}+\lambda (r_{e}^{*}-W_{e}x^{*}-b_{r}^{*})=0\\ J_{2}^{*}+\gamma (d_{x}^{*}-T_{x}x^{*}-b_{x}^{*})=0\\ J_{3}^{*}+\gamma (d_{y}^{*}-T_{y}x^{*}-b_{y}^{*})=0\\ b_{r}^{*}=b_{r}^{*}+(W_{e}x^{*}-r_{e}^{*})\\ b_{x}^{*}=b_{x}^{*}+(T_{x}x^{*}-d_{x}^{*})\\ b_{y}^{*}=b_{y}^{*}+(T_{y}x^{*}-d_{y}^{*}) \end{array}$$

From (A1) and (A3), we can see that $\left(x^{*},r_{e}^{*},d_{x}^{*},d_{y}^{*}\right)$ is a fixed point of the optimization problem (12) [36]. Subtracting the first equation of (A3) from the first equation of (A1), and then taking inner product operation with respect to $x_{}^{i+1}-x^{*}$ in both sides, we obtain

(A4) $$\begin{array}{l} \mu ||Rx_{ee}^{i+1}|{|}_{2}^{2}+\lambda ||W_{e}x_{ee}^{i+1}|{|}_{2}^{2}+\gamma ||T_{x}x_{ee}^{i+1}|{|}_{2}^{2}+\gamma ||T_{y}x_{ee}^{i+1}|{|}_{2}^{2}\\ \;-\lambda \langle r_{ee}^{i},W_{e}x_{ee}^{i+1}\rangle +\lambda \langle b_{re}^{i},W_{e}x_{ee}^{i+1}\rangle -\gamma \langle d_{xe}^{i},T_{x}x_{ee}^{i+1}\rangle \\ \;+\gamma \langle b_{xe}^{i},T_{x}x_{ee}^{i+1}\rangle -\gamma \langle d_{ye}^{i},T_{y}x_{ee}^{i+1}\rangle +\gamma \langle b_{ye}^{i},T_{y}x_{ee}^{i+1}\rangle \\ =0 \end{array}$$

where $x_{ee}^{i+1}=x_{}^{i+1}-x^{*}$, $r_{ee}^{i}=r_{e}^{i}-r_{e}^{*}$, $d_{xe}^{i}=d_{x}^{i}-d_{x}^{*}$, $d_{ye}^{i}=d_{y}^{i}-d_{y}^{*}$, $b_{re}^{i}=b_{r}^{i}-b_{r}^{*}$, $b_{xe}^{i}=b_{x}^{i}-b_{x}^{*}$, and $b_{ye}^{i}=b_{y}^{i}-b_{y}^{*}$. Next, subtracting the second equation of (A3) from the second equation of (A1), and then taking inner product operation with respect to $r_{e}^{i+1}-r_{e}^{*}$ in both sides, we obtain

(A5) $$\langle r_{ee}^{i+1},J_{1}^{i+1}-J_{1}^{*}\rangle +\lambda ||r_{ee}^{i+1}|{|}_{2}^{2}-\lambda \langle r_{ee}^{i+1},W_{e}x_{ee}^{i+1}\rangle -\lambda \langle r_{ee}^{i+1},b_{re}^{i}\rangle =0$$

Then, performing the above similar operations for the third equation to the seventh equation in (A3) and (A1), we have

(A6) $$\langle d_{xe}^{i+1},J_{2}^{i+1}-J_{2}^{*}\rangle +\gamma ||d_{xe}^{i+1}|{|}_{2}^{2}-\gamma \langle d_{xe}^{i+1},T_{x}x_{ee}^{i+1}\rangle -\gamma \langle d_{xe}^{i+1},b_{xe}^{i}\rangle =0$$

(A7) $$\langle d_{ye}^{i+1},J_{3}^{i+1}-J_{3}^{*}\rangle +\gamma ||d_{ye}^{i+1}|{|}_{2}^{2}-\gamma \langle d_{ye}^{i+1},T_{y}x_{ee}^{i+1}\rangle -\gamma \langle d_{ye}^{i+1},b_{ye}^{i}\rangle =0$$

(A8) $$b_{re}^{i+1}=b_{re}^{i}+(W_{e}x_{ee}^{i+1}-r_{ee}^{i+1})$$

(A9) $$b_{xe}^{i+1}=b_{xe}^{i}+(T_{x}x_{ee}^{i+1}-d_{xe}^{i+1})$$

(A10) $$b_{ye}^{i+1}=b_{ye}^{i}+(T_{y}x_{ee}^{i+1}-d_{ye}^{i+1})$$

where $d_{xe}^{i+1}=d_{x}^{i+1}-d_{x}^{*}$, $d_{ye}^{i+1}=d_{y}^{i+1}-d_{y}^{*}$, $b_{re}^{i+1}=b_{r}^{i+1}-b_{r}^{*}$, $b_{xe}^{i+1}=b_{x}^{i+1}-b_{x}^{*}$, $b_{ye}^{i+1}=b_{y}^{i+1}-b_{y}^{*}$. Summing the above Equations (A4)–(A7) gives

(A11) $$\begin{array}{l} \mu ||Rx_{ee}^{i+1}|{|}_{2}^{2}+\langle r_{ee}^{i+1},J_{1}^{i+1}-J_{1}^{*}\rangle \\ \;+\lambda \left(||W_{e}x_{ee}^{i+1}|{|}_{2}^{2}+||r_{ee}^{i+1}|{|}_{2}^{2}-\langle r_{ee}^{i+1}+r_{ee}^{i},W_{e}x_{ee}^{i+1}\rangle +\langle b_{re}^{i},W_{e}x_{ee}^{i+1}-r_{ee}^{i+1}\rangle \right)\\ \;+\langle d_{xe}^{i+1},J_{2}^{i+1}-J_{2}^{*}\rangle +\gamma \left(||T_{x}x_{ee}^{i+1}|{|}_{2}^{2}+||d_{xe}^{i+1}|{|}_{2}^{2}-\langle d_{xe}^{i+1}+d_{xe}^{i},T_{x}x_{ee}^{i+1}\rangle +\langle b_{xe}^{i},T_{x}x_{ee}^{i+1}-d_{xe}^{i+1}\rangle \right)\\ \;+\langle d_{ye}^{i+1},J_{3}^{i+1}-J_{3}^{*}\rangle +\gamma \left(||T_{y}x_{ee}^{i+1}|{|}_{2}^{2}+||d_{ye}^{i+1}|{|}_{2}^{2}-\langle d_{ye}^{i+1}+d_{ye}^{i},T_{y}x_{ee}^{i+1}\rangle +\langle b_{ye}^{i},T_{y}x_{ee}^{i+1}-d_{ye}^{i+1}\rangle \right)\\ =0 \end{array}$$

Taking the *l*_2_-norm on both sides of (A8), we obtain

(A12) $$\langle b_{re}^{i},W_{e}x_{ee}^{i+1}-r_{ee}^{i+1}\rangle =\frac{1}{2}\left(||b_{re}^{i+1}|{|}_{2}^{2}-||b_{re}^{i}|{|}_{2}^{2}-||W_{e}x_{ee}^{i+1}-r_{ee}^{i+1}|{|}_{2}^{2}\right)$$

Similarly, we can obtain

(A13) $$\langle b_{xe}^{i},T_{x}x_{ee}^{i+1}-d_{xe}^{i+1}\rangle =\frac{1}{2}\left(||b_{xe}^{i+1}|{|}_{2}^{2}-||b_{xe}^{i}|{|}_{2}^{2}-||T_{x}x_{ee}^{i+1}-d_{xe}^{i+1}|{|}_{2}^{2}\right)$$

(A14) $$\langle b_{ye}^{i},T_{y}x_{ee}^{i+1}-d_{ye}^{i+1}\rangle =\frac{1}{2}\left(||b_{ye}^{i+1}|{|}_{2}^{2}-||b_{ye}^{i}|{|}_{2}^{2}-||T_{y}x_{ee}^{i+1}-d_{ye}^{i+1}|{|}_{2}^{2}\right)$$

Substituting (A12)–(A14) into (A11), we obtain

(A15) $$\begin{array}{l} \frac{\lambda }{2}\left(||b_{re}^{i}|{|}_{2}^{2}-||b_{re}^{i+1}|{|}_{2}^{2}\right)+\frac{\gamma }{2}\left(||b_{xe}^{i}|{|}_{2}^{2}-||b_{xe}^{i+1}|{|}_{2}^{2}\right)+\frac{\gamma }{2}\left(||b_{ye}^{i}|{|}_{2}^{2}-||b_{ye}^{i+1}|{|}_{2}^{2}\right)\\ =\mu ||Rx_{ee}^{i+1}|{|}_{2}^{2}+\langle r_{ee}^{i+1},J_{1}^{i+1}-J_{1}^{*}\rangle \\ \;+\lambda \left(||W_{e}x_{ee}^{i+1}|{|}_{2}^{2}+||r_{ee}^{i+1}|{|}_{2}^{2}-\langle r_{ee}^{i+1}+r_{ee}^{i},W_{e}x_{ee}^{i+1}\rangle -\frac{1}{2}||W_{e}x_{ee}^{i+1}-r_{ee}^{i+1}|{|}_{2}^{2}\right)\\ \;+\langle d_{xe}^{i+1},J_{2}^{i+1}-J_{2}^{*}\rangle +\gamma \left(||T_{x}x_{ee}^{i+1}|{|}_{2}^{2}+||d_{xe}^{i+1}|{|}_{2}^{2}-\langle d_{xe}^{i+1}+d_{xe}^{i},T_{x}x_{ee}^{i+1}\rangle -\frac{1}{2}||T_{x}x_{ee}^{i+1}-d_{xe}^{i+1}|{|}_{2}^{2}\right)\\ \;+\langle d_{ye}^{i+1},J_{3}^{i+1}-J_{3}^{*}\rangle +\gamma \left(||T_{y}x_{ee}^{i+1}|{|}_{2}^{2}+||d_{ye}^{i+1}|{|}_{2}^{2}-\langle d_{ye}^{i+1}+d_{ye}^{i},T_{y}x_{ee}^{i+1}\rangle -\frac{1}{2}||T_{y}x_{ee}^{i+1}-d_{ye}^{i+1}|{|}_{2}^{2}\right)\\ =\mu ||Rx_{ee}^{i+1}|{|}_{2}^{2}+\langle r_{ee}^{i+1},J_{1}^{i+1}-J_{1}^{*}\rangle +\frac{\lambda }{2}\left(||r_{ee}^{i+1}|{|}_{2}^{2}-||r_{ee}^{i}|{|}_{2}^{2}+||W_{e}x_{ee}^{i+1}-r_{ee}^{i}|{|}_{2}^{2}\right)\\ \;+\langle d_{xe}^{i+1},J_{2}^{i+1}-J_{2}^{*}\rangle +\frac{\gamma }{2}\left(||d_{xe}^{i+1}|{|}_{2}^{2}-||d_{xe}^{i}|{|}_{2}^{2}+||T_{x}x_{ee}^{i+1}-d_{xe}^{i}|{|}_{2}^{2}\right)\\ \;+\langle d_{ye}^{i+1},J_{3}^{i+1}-J_{3}^{*}\rangle +\frac{\gamma }{2}\left(||d_{ye}^{i+1}|{|}_{2}^{2}-||d_{ye}^{i}|{|}_{2}^{2}+||T_{y}x_{ee}^{i+1}-d_{ye}^{i}|{|}_{2}^{2}\right) \end{array}$$

By summing up (A15) from 0 to *K*, we have

(A16) $$\begin{array}{l} \frac{\lambda }{2}\left(||b_{re}^{0}|{|}_{2}^{2}-||b_{re}^{K+1}|{|}_{2}^{2}\right)+\frac{\gamma }{2}\left(||b_{xe}^{0}|{|}_{2}^{2}-||b_{xe}^{K+1}|{|}_{2}^{2}\right)+\frac{\gamma }{2}\left(||b_{ye}^{0}|{|}_{2}^{2}-||b_{ye}^{K+1}|{|}_{2}^{2}\right)\\ =\mu \sum_{i=0}^{K}||Rx_{ee}^{i+1}|{|}_{2}^{2}+\sum_{i=0}^{K}\langle r_{ee}^{i+1},J_{1}^{i+1}-J_{1}^{*}\rangle +\frac{\lambda }{2}\left(\sum_{i=0}^{K}||W_{e}x_{ee}^{i+1}-r_{ee}^{i}|{|}_{2}^{2}+||r_{ee}^{K+1}|{|}_{2}^{2}-||r_{ee}^{0}|{|}_{2}^{2}\right)\\ \;+\sum_{i=0}^{K}\langle d_{xe}^{i+1},J_{2}^{i+1}-J_{2}^{*}\rangle +\frac{\gamma }{2}\left(\sum_{i=0}^{K}||T_{x}x_{ee}^{i+1}-d_{xe}^{i}|{|}_{2}^{2}+||d_{xe}^{K+1}|{|}_{2}^{2}-||d_{xe}^{0}|{|}_{2}^{2}\right)\\ \;+\sum_{i=0}^{K}\langle d_{ye}^{i+1},J_{3}^{i+1}-J_{3}^{*}\rangle +\frac{\gamma }{2}\left(\sum_{i=0}^{K}||T_{y}x_{ee}^{i+1}-d_{ye}^{i}|{|}_{2}^{2}+||d_{ye}^{K+1}|{|}_{2}^{2}-||d_{ye}^{0}|{|}_{2}^{2}\right) \end{array}$$

From (A16), we can obtain

(A17) $$\begin{array}{l} \frac{\lambda }{2}\left(||b_{re}^{0}|{|}_{2}^{2}+||r_{ee}^{0}|{|}_{2}^{2}\right)+\frac{\gamma }{2}\left(||b_{xe}^{0}|{|}_{2}^{2}+||d_{xe}^{0}|{|}_{2}^{2}\right)+\frac{\gamma }{2}\left(||b_{ye}^{0}|{|}_{2}^{2}+||d_{ye}^{0}|{|}_{2}^{2}\right)\\ \geq \mu \sum_{i=0}^{K}||Rx_{ee}^{i+1}|{|}_{2}^{2}+\sum_{i=0}^{K}\langle r_{ee}^{i+1},J_{1}^{i+1}-J_{1}^{*}\rangle +\frac{\lambda }{2}\left(\sum_{i=0}^{K}||W_{e}x_{ee}^{i+1}-r_{ee}^{i}|{|}_{2}^{2}+||r_{ee}^{K+1}|{|}_{2}^{2}\right)\\ \;+\sum_{i=0}^{K}\langle d_{xe}^{i+1},J_{2}^{i+1}-J_{2}^{*}\rangle +\frac{\gamma }{2}\left(\sum_{i=0}^{K}||T_{x}x_{ee}^{i+1}-d_{xe}^{i}|{|}_{2}^{2}+||d_{xe}^{K+1}|{|}_{2}^{2}\right)\\ \;+\sum_{i=0}^{K}\langle d_{ye}^{i+1},J_{3}^{i+1}-J_{3}^{*}\rangle +\frac{\gamma }{2}\left(\sum_{i=0}^{K}||T_{y}x_{ee}^{i+1}-d_{ye}^{i}|{|}_{2}^{2}+||d_{ye}^{K+1}|{|}_{2}^{2}\right) \end{array}$$

As $||r_{e}^{}|{|}_{1}$, $||d_{x}^{}|{|}_{1}$ and $||d_{y}^{}|{|}_{1}$ are convex functions, it has been proven that $\langle r_{ee}^{i+1},J_{1}^{i+1}-J_{1}^{*}\rangle \geq 0$, $\langle d_{xe}^{i+1},J_{2}^{i+1}-J_{2}^{*}\rangle \geq 0$ and $\langle d_{ye}^{i+1},J_{3}^{i+1}-J_{3}^{*}\rangle \geq 0$ in [30]. Therefore, the above inequality implies that

(A18) $$\underset{i\to \infty }{\lim}||Rx_{ee}^{i}|{|}_{2}^{2}=\underset{i\to \infty }{\lim}||Rx_{}^{i}-Rx_{}^{*}|{|}_{2}^{2}=\underset{i\to \infty }{\lim}\langle R^{T}(Rx_{}^{i}-Rx_{}^{*}),x_{}^{i}-x_{}^{*}\rangle =0$$

(A19) $$\underset{i\to \infty }{\lim}\langle r_{ee}^{i},J_{1}^{i}-J_{1}^{*}\rangle =0$$

(A20) $$\underset{i\to \infty }{\lim}\langle d_{xe}^{i},J_{2}^{i}-J_{2}^{*}\rangle =0$$

(A21) $$\underset{i\to \infty }{\lim}\langle d_{ye}^{i},J_{3}^{i}-J_{3}^{*}\rangle =0$$

Then, considering the Bregman distance here [25]

(A22) $$D_{E}^{P}(u,v)=E(u)-E(v)-\langle P,u-v\rangle$$

where *P* is in the subgradient of the convex function *E* at *v*, and $D_{E}^{P}(u,v)\geq 0$. Let convex function $E(x)=\frac{1}{2}||Rx-y|{|}_{2}^{2}$. By (A22), we have

(A23) $$\begin{array}{l} D_{E}^{P}(x^{i},x^{*})+D_{E}^{P^{\prime }}(x^{*},x^{i})\\ =\langle P-P^{\prime },x^{i}-x^{*}\rangle \\ =\langle R^{T}(Rx_{}^{i}-Rx_{}^{*}),x^{i}-x^{*}\rangle \end{array}$$

As $D_{E}^{P}(x^{i},x^{*})\geq 0$ and $D_{E}^{P^{\prime }}(x^{*},x^{i})\geq 0$ [28], Equations (A18) and (A23) imply that

(A24) $$\underset{i\to \infty }{\lim}D_{E}^{P}(x^{i},x^{*})=\underset{i\to \infty }{\lim}\left(\frac{1}{2}||Rx^{i}-y|{|}_{2}^{2}-\frac{1}{2}||Rx^{*}-y|{|}_{2}^{2}-\langle R^{T}(Rx_{}^{i}-Rx_{}^{*}),x^{i}-x^{*}\rangle \right)=0$$

Let convex function $E(r_{e})=||r_{e}|{|}_{1}$. By (A22), we have

(A25) $$\begin{array}{l} D_{E}^{J_{1}^{i}}(r_{e}^{i},r_{e}^{*})+D_{E}^{J_{1}^{*}}(r_{e}^{*},r_{e}^{i})\\ =\langle J_{1}^{i}-J_{1}^{*},r_{e}^{i}-r_{e}^{*}\rangle \\ =\langle J_{1}^{i}-J_{1}^{*},r_{ee}^{i}\rangle \end{array}$$

As $D_{E}^{J_{1}}(r_{e}^{i},r_{e}^{*})\geq 0$ and $D_{E}^{{J^{\prime }}_{1}}(r_{e}^{*},r_{e}^{i})\geq 0$ [28], Equations (A19) and (A25) imply that

(A26) $$\begin{array}{l} \underset{i\to \infty }{\lim}D_{E}^{J_{1}}(r_{e}^{i},r_{e}^{*})\\ =\underset{i\to \infty }{\lim}\left(||r_{e}^{i}|{|}_{1}-||r_{e}^{*}|{|}_{1}-\langle r_{ee}^{i},J_{1}^{*}\rangle \right)\\ =\underset{i\to \infty }{\lim}\left(||W_{e}x_{}^{i}|{|}_{1}-||W_{e}x_{}^{*}|{|}_{1}-\langle W_{e}(x_{}^{i}-x_{}^{*}),J_{1}^{*}\rangle \right)\\ =0 \end{array}$$

Similarly, we have

(A27) $$\underset{i\to \infty }{\lim}\left(||T_{x}x_{}^{i}|{|}_{1}-||T_{x}x^{*}|{|}_{1}-\langle T_{x}(x_{}^{i}-x^{*}),J_{2}^{*}\rangle \right)=0$$

(A28) $$\underset{i\to \infty }{\lim}\left(||T_{y}x_{}^{i}|{|}_{1}-||T_{y}x^{*}|{|}_{1}-\langle T_{y}(x_{}^{i}-x^{*}),J_{3}^{*}\rangle \right)=0$$

By (A24), (A26)–(A28), we obtain

(A29) $$\begin{array}{l} \underset{i\to \infty }{\lim}(\frac{\mu }{2}||Rx^{i}-y|{|}_{2}^{2}-\frac{\mu }{2}||Rx^{*}-y|{|}_{2}^{2}-\mu \langle R^{T}(Rx_{}^{i}-Rx_{}^{*}),x^{i}-x^{*}\rangle +||W_{e}x_{}^{i}|{|}_{1}-||W_{e}x_{}^{*}|{|}_{1}\\ \;\;-\langle W_{e}(x_{}^{i}-x_{}^{*}),J_{1}^{*}\rangle +||T_{x}x_{}^{i}|{|}_{1}-||T_{x}x^{*}|{|}_{1}-\langle T_{x}(x_{}^{i}-x^{*}),J_{2}^{*}\rangle \\ \;\;+||T_{y}x_{}^{i}|{|}_{1}-||T_{y}x^{*}|{|}_{1}-\langle T_{y}(x_{}^{i}-x^{*}),J_{3}^{*}\rangle )\\ =\underset{i\to \infty }{\lim}(||W_{e}x_{}^{i}|{|}_{1}-||W_{e}x_{}^{*}|{|}_{1}+||T_{x}x_{}^{i}|{|}_{1}-||T_{x}x^{*}|{|}_{1}+||T_{y}x_{}^{i}|{|}_{1}-||T_{y}x^{*}|{|}_{1}+\frac{\mu }{2}||Rx^{i}-y|{|}_{2}^{2}\\ \;\;-\frac{\mu }{2}||Rx^{*}-y|{|}_{2}^{2}-\langle x^{i}-x^{*},\mu R^{T}(Rx_{}^{i}-Rx_{}^{*})+W_{e}J_{1}^{*}+T_{x}J_{2}^{*}+T_{y}J_{3}^{*}\rangle )\\ =\underset{i\to \infty }{\lim}(||W_{e}x_{}^{i}|{|}_{1}-||W_{e}x_{}^{*}|{|}_{1}+||T_{x}x_{}^{i}|{|}_{1}-||T_{x}x^{*}|{|}_{1}+||T_{y}x_{}^{i}|{|}_{1}-||T_{y}x^{*}|{|}_{1}\\ \;\;+\frac{\mu }{2}||Rx^{i}-y|{|}_{2}^{2}-\frac{\mu }{2}||Rx^{*}-y|{|}_{2}^{2})\\ =0 \end{array}$$

where the second equality in (A29) is obtained by applying (A2). Therefore, the convergence theorem for (27) has been proven. □

## References

[1] Mittleman D.M. (2018). Twenty years of terahertz imaging. Opt. Express.

[2] Zanotto L., Piccoli R., Dong J., Morandotti R., Razzari L. (2020). Single-pixel terahertz imaging: A review. Opto-Electron. Adv..

[3] Stantchev R.I., Yu X., Blu T., Pickwell-MacPherson E. (2020). Real-time terahertz imaging with a single-pixel detector. Nat. Commun..

[4] Park H., Son J.-H., Ahn C.-B. (2016). Enhancement of terahertz reflection tomographic imaging by interference cancellation between layers. Opt. Express.

[5] Zhai M., Locquet A., Citrin D. (2020). Pulsed THz imaging for thickness characterization of plastic sheets. NDT E Int..

[6] Redo-Sanchez A., Heshmat B., Aghasi A., Naqvi S., Zhang M., Romberg J., Raskar R. (2016). Terahertz time-gated spectral imaging for content extraction through layered structures. Nat. Commun..

[7] Wang Q., Zhou H., Xia R., Liu Q., Zhao B.Y. (2020). Time Segmented Image Fusion Based Multi- Depth Defects Imaging Method in Composites With Pulsed Terahertz. IEEE Access.

[8] Gowen A., O’Sullivan C., O’Donnell C. (2012). Terahertz time domain spectroscopy and imaging: Emerging techniques for food process monitoring and quality control. Trends Food Sci. Technol..

[9] Guerboukha H., Nallappan K., Skorobogatiy M. (2018). Toward real-time terahertz imaging. Adv. Opt. Photonics.

[10] D’Arco A., Di Fabrizio M., Dolci V., Petrarca M., Lupi S. (2020). THz Pulsed Imaging in Biomedical Applications. Condens. Matter.

[11] Park J.Y., Choi H.J., Cheon H., Cho S.W., Lee S., Son J.-H. (2017). Terahertz imaging of metastatic lymph nodes using spectroscopic integration technique. Biomed. Opt. Express.

[12] Jiang Z., Zhang X.-C. (1999). Terahertz imaging via electrooptic effect. IEEE Trans. Microw. Theory Tech..

[13] Xu J., Zhang X.-C. (2006). Terahertz wave reciprocal imaging. Appl. Phys. Lett..

[14] Chan W.L., Moravec M.L., Baraniuk R.G., Mittleman D. (2008). Terahertz imaging with compressed sensing and phase retrieval. Opt. Lett..

[15] Chan W.L., Charan K., Takhar D., Kelly K.F., Baraniuk R.G., Mittleman D. (2008). A single-pixel terahertz imaging system based on compressed sensing. Appl. Phys. Lett..

[16] Zhang Z., Ma X., Zhong J. (2015). Single-pixel imaging by means of Fourier spectrum acquisition. Nat. Commun..

[17] Yee D.-S., Jin K.H., Yahng J.S., Yang H.-S., Kim C.Y., Ye J.C. (2015). High-speed terahertz reflection three-dimensional imaging using beam steering. Opt. Express.

[18] Lu Y., Wang X.-K., Sun W.-F., Feng S.-F., Ye J.-S., Han P., Zhang Y. (2020). Reflective Single-Pixel Terahertz Imaging Based on Compressed Sensing. IEEE Trans. Terahertz Sci. Technol..

[19] Cho S.-H., Lee S.-H., Nam-Gung C., Oh S.-J., Son J.-H., Park H., Ahn C.-B. (2011). Fast terahertz reflection tomography using block-based compressed sensing. Opt. Express.

[20] Hwang B.-M., Lee S.H., Lim W.-T., Ahn C.-B., Son J.-H., Park H. (2011). A Fast Spatial-domain Terahertz Imaging Using Block-based Compressed Sensing. J. Infrared Millim. Terahertz Waves.

[21] Candes E.J., Wakin M.B., Boyd S.P. (2008). Enhancing Sparsity by Reweighted l(1) Minimization. J. Fourier Anal. Appl..

[22] Wang Y., Yang J., Yin W., Zhang Y. (2008). A New Alternating Minimization Algorithm for Total Variation Image Reconstruction. SIAM J. Imaging Sci..

[23] Gonzalez R.C., Woods R.E. (2007). Digital Image Processing.

[24] Zhang Y., Peterson B.S., Dong Z. Increasing sparsity in compressed sensing MRI by exponent of wavelet coefficients. Proceedings of the 19th Annual Meeting of ISMRM.

[25] Goldstein T., Osher S. (2009). The Split Bregman Method for L1-Regularized Problems. SIAM J. Imaging Sci..

[26] Li L., Xiao S., Zhao Y. (2020). Image Compressive Sensing via Hybrid Nonlocal Sparsity Regularization. Sensors.

[27] Yin W., Osher S., Goldfarb D., Darbon J. (2008). Bregman iterative algorithms for l1-minimization with applications to compressed sensing. SIAM J. Imaging Sci..

[28] Cai J.-F., Osher S., Shen Z. (2009). Linearized Bregman iterations for compressed sensing. Math. Comput..

[29] Cai J.-F., Osher S., Shen Z. (2009). Linearized Bregman Iterations for Frame-Based Image Deblurring. SIAM J. Imaging Sci..

[30] Zou J., Li H., Li G. (2018). Split Bregman Algorithm for Structured Sparse Reconstruction. IEEE Access.

[31] Taylor Z.D., Singh R.S., Culjat M.O., Suen J.Y., Grundfest W.S., Lee H., Brown E.R. (2008). Reflective terahertz imaging of porcine skin burns. Opt. Lett..

[32] Jiang Y., Ge H., Lian F., Zhang Y., Xia S. (2015). Discrimination of moldy wheat using terahertz imaging combined with multivariate classification. RSC Adv..

[33] Kim K.W., Kim K.S., Kim H., Lee S.H., Park J.H., Han J.H., Seok S.H., Park J., Choi Y., Kim Y.I. (2012). Terahertz dynamic imaging of skin drug absorption. Opt. Express.

[34] Karpowicz N., Zhong H., Xu J., Lin K.-I., Hwang J.-S., Zhang X.-C. (2005). Comparison between pulsed terahertz time-domain imaging and continuous wave terahertz imaging. Semicond. Sci. Technol..

[35] Tewari P. (2013). Reflective Terahertz Imaging for Early Diagnosis of Skin Burn Severity.

[36] Hale E.T., Yin W.T., Zhang Y. (2008). A fixed-point continuation method for l1-regularized minimization with applications to compressed sensing. SIAM J. Optim..

